# Supplementary figures and images for: Characterization of locomotor phenotypes in zebrafish larvae requires testing under both light and dark conditions
Source: PLoS One. 2022 Apr 1;17(4):e0266491. doi: 10.1371/journal.pone.0266491 (PMC8974968; doi:10.1371/journal.pone.0266491)

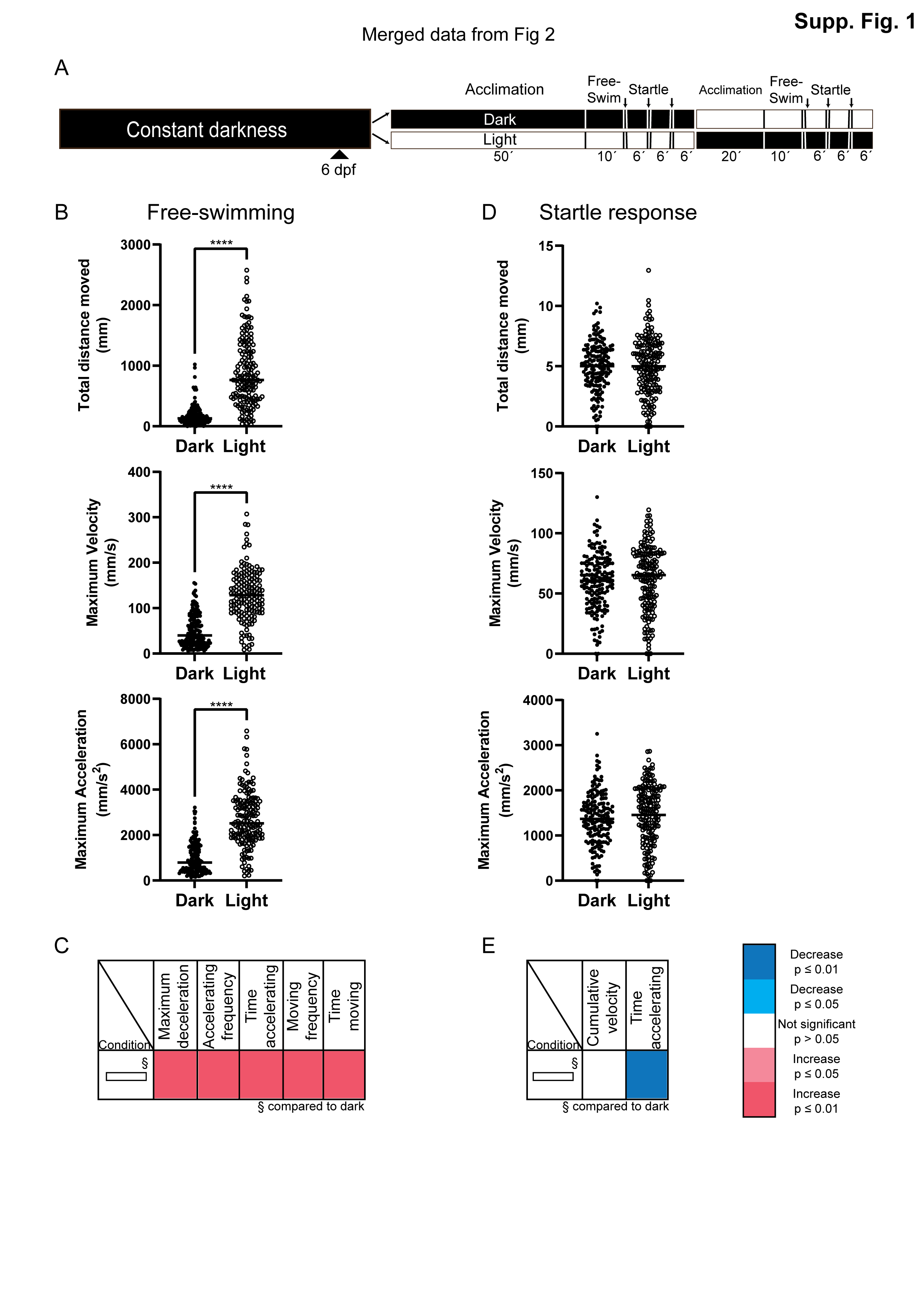

Supplement: S1 Fig — Merging of data from the same illumination following sequential testing of light and dark. (A) Experimental scheme of sequential application of illumination, light (open circles; white) and dark (solid circles; black). (B, C) Under light condition, larvae performed higher activity in free-swimming episode. (D, E) The differences were disappeared in terms of startle response. Free-swimming: n = 162–163 per group, startle response: n = 172 per group. Data is presented in scatterplots showing individual values and group mean or median. Analysis was performed with paired t-tests. Significance: *p ≤ 0.05, **p ≤ 0.01, ***p ≤ 0.001, ****p ≤ 0.0001. (TIF) [file pone.0266491.s001.tif]

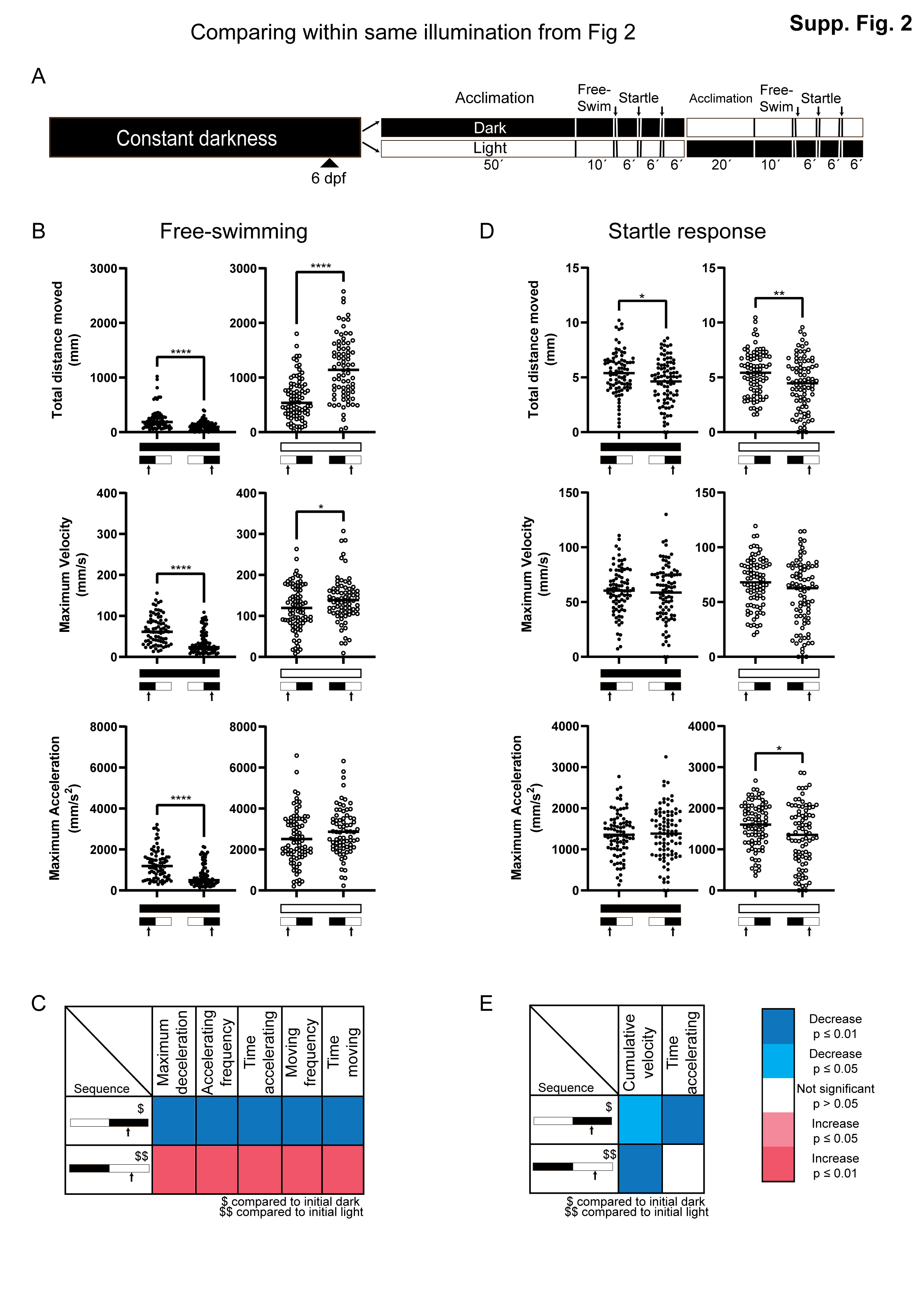

Supplement: S2 Fig — Comparison of data from the same illumination following sequential testing of light and dark. (A) Experimental scheme of sequential application of illumination, light (open circles; white) and dark (solid circles; black). (B, C) In terms of free-swimming, second dark and second light condition had different strength compared to first order same illumination. (D, E) Regardless of illumination, second condition caused a decrease in startle response parameters. Free-swimming: n = 76–86 per group, startle response: n = 85–87 per group. Data is presented in scatterplots showing individual values and group mean or median. Analysis was performed with paired t-tests. Significance: *p ≤ 0.05, **p ≤ 0.01, ***p ≤ 0.001, ****p ≤ 0.0001. (TIF) [file pone.0266491.s002.tif]

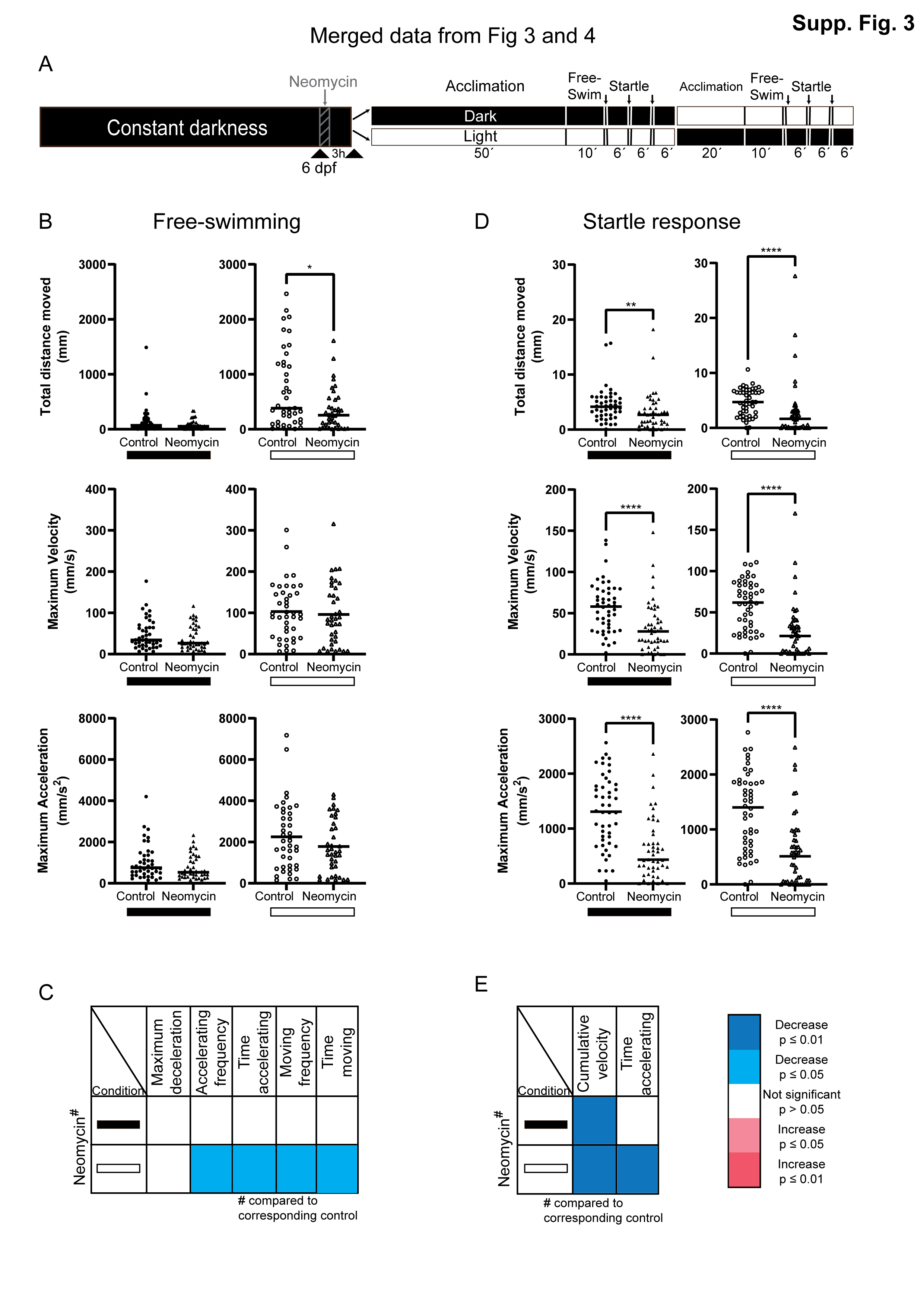

Supplement: S3 Fig — Merging of data from the same treatment (control vs neomycin) under similar illumination following sequential testing of light and dark. (A) Experimental scheme of sequential application of illumination, light (open circles/triangles; white) and dark (solid circles/triangles; black), after neomycin treatment (triangles). (B, C) All significances were disappeared under dark condition. (D, E) The significant differences were remained under both dark and light condition in case of startle response. Free-swimming: n = 35–39 per group, startle response: n = 47–48 per group. Data is presented in scatterplots showing individual values and group mean or median. Significance: *p ≤ 0.05, **p ≤ 0.01, ***p ≤ 0.001, ****p ≤ 0.0001. (TIF) [file pone.0266491.s003.tif]
