# Supplementary material for: Characterization of locomotor phenotypes in zebrafish larvae requires testing under both light and dark conditions
Source: PLoS One. 2022 Apr 1;17(4):e0266491. doi: 10.1371/journal.pone.0266491 (PMC8974968; doi:10.1371/journal.pone.0266491)
Supplement: Attachment — Submitted filename: Response to Reviewers.docx [file pone.0266491.s006.docx]

**Editorial comments:**

1. Please ensure that your manuscript meets PLOS ONE's style requirements, including those for file naming. The PLOS ONE style templates can be found at 
<https://journals.plos.org/plosone/s/file?id=wjVg/PLOSOne_formatting_sample_main_body.pdf> and 
<https://journals.plos.org/plosone/s/file?id=ba62/PLOSOne_formatting_sample_title_authors_affiliations.pdf>

Response: We thank the Editor for this recall. Now manuscript and additional files should meet PLOS ONE’s style requirements.

2. We note that the grant information you provided in the ‘Funding Information’ and ‘Financial Disclosure’ sections do not match.

When you resubmit, please ensure that you provide the correct grant numbers for the awards you received for your study in the ‘Funding Information’ section.

Response: We thank the Editor. We addressed the concerns about “Funding Information” section.

3. Please review your reference list to ensure that it is complete and correct. If you have cited papers that have been retracted, please include the rationale for doing so in the manuscript text, or remove these references and replace them with relevant current references. Any changes to the reference list should be mentioned in the rebuttal letter that accompanies your revised manuscript. If you need to cite a retracted article, indicate the article’s retracted status in the References list and also include a citation and full reference for the retraction notice.

Response: We thank the Editor for noticing. We reviewed the reference list; a book title was missing for reference [1] and some new references were added to list. Now it is complete and should be correct. None of papers in the reference list was retracted at the resubmission time.

4. While revising your submission, please upload your figure files to the Preflight Analysis and Conversion Engine (PACE) digital diagnostic tool, <https://pacev2.apexcovantage.com/>. PACE helps ensure that figures meet PLOS requirements. To use PACE, you must first register as a user. Registration is free. Then, login and navigate to the UPLOAD tab, where you will find detailed instructions on how to use the tool. If you encounter any issues or have any questions when using PACE, please email PLOS at [figures@plos.org](mailto:figures@plos.org). Please note that Supporting Information files do not need this step.

Response: We thank the Editor and have uploaded and checked Figures with PACE.

**Reviewers' comments:**

We would like to thank both reviewers for their efforts and constructive comments. We amended our manuscript according to their suggestions. Please find a detailed response below.

**Reviewer #1:**

Dear author,
Thank you for the very interesting manuscript.
It is very important to know the fish corrections for behavioral analytic.

I'll list below what I was wondering about.

1. Line 53-54 I get the message "incorrect illumination was used", what's "incorrect"? The behavior of the fish depends on the experimental conditions, and the results will vary depending on the conditions different from your own experimental conditions. Researchers never call it a "incorrect".

Response: The authors agree with the Reviewer and have replaced that statement with “because one illumination condition was neglected” in line 54-55.

2.Why did you use "tap" for escape stimulation? If you look at the graph, you can see that there is no extreme escape under any conditions. We also need to explain the "tap" method in more detail.

Response: The tapping device is an add-on property of the Daniovision observation chamber. It is used to evoke startle response (van den Bos et al., 2017; Faria et al., 2019; Han et al., 2020). To be more correct, we have changed escape responses with startle responses throughout revised manuscript.

We have already checked distance moved, maximum velocity values for whole experimental duration with 1-min time bins. The time bin graphs (data not shown in the manuscript) demonstrated that at each tapping stimulus, distance moved and maximum velocity were increased 4-5 times compared to baseline free-swimming behavior and also compared to resting time between tapping stimuli similar to data shown in van den Bos et al., 2017. Each group had response to tapping stimulus compared to their own baseline behavior but that comparisons did not include in the present work since our aim is to unravel the behaviors of larvae under different conditions including housing, illumination and neuromast ablation.

In Materials and Methods, line 122-124, we explained the tapping mechanism and details in our experimental setup.

3.Zebrafish are known to swim towards light (Wolf et al., 2017). Therefore, when evaluating the travel distance in a certain time unit, it is natural that the travel distance will be longer when it is bright. Repeated light-dark stimuli aim to conduct experiments more efficiently by measuring the distance traveled in a short time using the escape behavior of zebrafish. I was not sure about this experimental design and conclusion. I think it would be better to summarize it a little more concisely.

Response: In our experiments, we don`t have illumination differences within the arena, entire arena is under either dark or light.

Moreover, we did not use light as a startle response because we wanted to assess differences between these illumination settings independently. Therefore, we used the tapping device incorporated into the Daniovision chamber. We are unsure about the comment “measuring the distance traveled in a short time the escape behavior of zebrafish” because we believe this is exactly what we performed during our escape (startle) response analysis (time analyzed is 280 ms after tapping).

4. The number of zebrafish juveniles used in the experiment varies. In such cases, you should use a "scatter plot" instead of a bar chart. You guys use "prism" for statistical processing, so I think it's easy to do. Show me once, whether or not you use a "scatter plot".

Response: The authors agree and thank the Reviewer for the comment. The bar graphs were replaced with scatterplots for each figure. Also, the sentence in Material and Methods, line 149-151 were rewritten, also figure legends were adjusted accordingly.

**Reviewer #2:**

The authors have characterised changes in free-swimming and escape response of larval zebrafish under different rearing protocols and light illumination. The manuscript has a sound research question, is written is a clear english and well-exposes methods and results. The discussion of results is also appropriate. The study adds an important piece of information to the literature with regards to the behavioural phenotype of larval zebrafish as a consequence of variability in illumination.

I suggest a minor revision, following the comments below:

Typos:
- in the abstract, line 22: please remove one of the 2 “only” - it’s repeated twice.
- in the abstract, line 23: I suggest rephrasing “one illumination setting” with “the neglected illumination setting” for better clarity
- in the abstract, line 27: please remove “display” - reveal is sufficient.
- Line 253: Please remove the full stop in this phrase “…..striped bar graphs). had……“

Response: We thank the Reviewer for bringing these typos to our attention. They have been corrected in the revised manuscript.

Comments:-
- Please explain more in the discussion section (with more reference to the habituation literature) why the escape response under light conditions was weaker. E.g., exposure to daylight induces long-term habituation to light, reducing the escape response to light stimuli. Do the authors believe this was a sensory or central mechanism of habituation?

Response: In the present study, light is not utilized to evoke escape (startle) response, instead we used mechanical/acoustic startle response by a tapping mechanism. The main purpose was to understand the behavior of fish under different, independent, illumination settings as to not miss any phenotype (Fig 1). The behavior of larvae tested under light conditions were different than larvae tested under dark conditions, as shown in the data represented with Fig 1. Thus we are not sure what the reviewer meant with “exposure to daylight induces long-term habituation to light, reducing the escape response to light stimuli”. Escape (startle) response to tapping stimuli under light condition was weaker compared to response to tapping stimuli when tested under dark condition, for larvae were housed under a day/night cycle prior to behavioral assessment.

We do agree with the reviewer´s comment that additional words should have been devoted to the habituation effect and the light/dark differences observed. To this extend, we have added the habituation definition in line 335-353. Also, the discussion regarding a stronger startle response under darkness in line 365-366 (335-353) was expanded.

- The authors stated that larvae under a day/night cycle showed a weaker escape response in light conditions. However, it is now stated whether this is meant in terms of frequency/count of escape responses, or in terms of distance travelled (mm) ?

Response: In the revised manuscript we now clarify/specify the parameters that are weaker under light conditions: line 28-30.
